# Spatiotemporal analysis of *Anopheles gambiae* larval sites and malaria transmission in Djilakh, Central Senegal

**DOI:** 10.1186/s12936-025-05388-5

**Published:** 2025-05-26

**Authors:** Assane Ndiaye, Camille Morlighem, Aminata Niang Diène, Moussa Kane, Abdoulaye Diallo, Fassiatou Tairou, Mohamed Abderemane Nourdine, Pape Cheikh Sarr, Lassana Konaté, Ousmane Faye, Oumar Gaye, El Hadji Amadou Niang, Catherine Linard, Ousmane Sy

**Affiliations:** 1 Faculté des Lettres et Sciences Humaines, Département de Géographie, Université Cheikh Anta Diop, Dakar, Sénégal; 2 Faculté des Sciences et Techniques, Laboratoire d’Ecologie Vectorielle et Parasitaire, Université Cheikh Anta Diop, Dakar, Sénégal; 3 Faculté de Médecine, Laboratoire de Parasitologie Médicale, Pharmacie et d’Odonto-stomatologie, MARCAD Programme, Université Cheikh Anta Diop, Dakar, Sénégal; 4 Department of Geography, University of Namur, 5000 Namur, Belgium; 5 ILEE University of Namur, 5000 Namur, Belgium; 6 NAmur Research Institute for LIfe Sciences (NARILIS), University of Namur, 5000 Namur, Belgium; 7 Computer Science Section, Department of Mathematics and Computer Science at UCAD, Université Cheikh Anta Diop, Dakar, Sénégal

**Keywords:** Mapping, Breeding sites, Malaria risk, Hotspots, Senegal

## Abstract

**Background:**

The progress made against malaria has resulted in a nationwide reduction of the disease burden in Senegal. The observed overall low transmission levels are, however, marked by an important spatial heterogeneity with hotspots subsisting in several parts of the country. This requires the determination of the local and regional factors of the observed disparities for tailored interventions to accelerate malaria elimination everywhere. This study aimed to demonstrate the role of larval breeding sites on malaria epidemiological trends in Djilakh, which is one of the malaria hotspots of the Mbour health district.

**Methods:**

This study was carried out between 2013 and 2017, during the rainy season (June-November) of each year and surveys per year. The malaria incidence consisted of cases confirmed by RDT and climate data, including the rainfall were retrieved from the Mbour weather station. To assess the impact of larval breeding sites on malaria transmission in Djilakh village, logistic regression under the Poisson models were run. The QGIS 2.2.0 free mapping software was used to generate maps.

**Results:**

The results showed that mosquito breeding sites found within and in the vicinity of the study village consisted of natural temporary ponds, characterized by clay and clay-sandy soils. The analysis of meteorological and malaria morbidity indicated that malaria transmission is influenced by precipitation. The correlation between malaria morbidity and functioning breeding sites varied throughout the rainy season, depending on the size and stability of the existing breeding sites. The incidence of malaria cases was significantly higher (82.4%; 103/125; P < 0.011; OR = 27.006) in hamlets closer to the breeding sites (less than 500 m), declining gradually with distance with 17.6% (22/125) of the cases recorded in hamlets located between 500 and 1000 m apart from the larval habitats and, no cases in the most remote hamlets (> 1000 m).

**Conclusions:**

These findings represent a preliminary step towards a better understanding of how the environmental factors influence the persistence of malaria transmission in the studied hotspot villages in Senegal. The generated results indicate a need for targeted control actions in the studied site.

## Background

Malaria is a vector-borne disease transmitted by *Anopheles* mosquitoes. Despite all the efforts to control malaria, it remains a major public health problem worldwide, especially in underdeveloped countries. *Plasmodium falciparum* is the primary parasite of human malaria, accounting for 99.7% of the estimated cases across sub-Saharan Africa, 71% in the Eastern Mediterranean Region, 65% in the Western Pacific Region and 50% of cases in the South-East Asia Region [1]. According to the 2024 World Malaria report, 263 million cases were estimated from 83 malaria-endemic countries and territories in 2023, thus representing an increase of 11 million cases compared to 2022 [2].

In Senegal, malaria remains the leading cause of consultations, with approximately 358,033 confirmed cases and 273 deaths in 2020, mainly among pregnant women and children under 5 [3]. Malaria is endemic with significant variabilities across the country. The incidence is the highest in the so-called red zone, which encompasses the three southern regions of Kolda, Tambacounda and Kédougou which accounted for 83.3% of the malaria cases in 2020 [4]. On the other hand, the western-central region is characterized by lower but heterogeneous malaria burden following the successful implementation of integrated malaria control interventions over the last few years, including the seasonal malaria chemoprevention and indoor residual spraying, which resulted in the global decline of malaria in the whole region, but the persisting in few hotspots, where residual transmission still ongoing [5].

In Senegal, 21 anopheline species have been described so far [6, 7]. Of these, the *Anopheles gambiae* complex (*Anopheles arabiensis, Anopheles melas, Anopheles gambiae* and *Anopheles coluzzii*) and *Anopheles funestus*, are the main vectors of malaria, contributing at different levels to the transmission of the disease. Therefore, understanding the factors influencing their biology and thus their importance in the transmission is crucial for tailored vector control interventions against malaria.

The effective implementation of control strategies requires appropriate identification and understanding of local epidemiological dynamics and environmental factors associated with a specific geographical area. Specifically, when the current control effort of the disease is hindered by the lack of information about how breeding sites function and may shape local transmission dynamics. Indeed, the mineralogical composition of soils can influence the number of mosquito breeding sites, particularly when combined with high rainfall [8]. Some studies have indicated that the dynamics of anopheline mosquito breeding sites, in particular their abundance, stability and proximity to human habitation, have a significant impact on the disease transmission in some villages of central-western Senegal [9]. Larval development depends on a number of parameters, including physical and chemical characteristics and climatic factors [10–12]. An improved understanding of the factors influencing the development of *Anopheles* larvae within their breeding sites may facilitate more effective decision-making among those responsible for public health.

The objective of this study is to identify the environmental factors influencing the proliferation of malaria vectors in the hotspot village of Djilakh, in central Senegal over the time. The data generated will contribute to a better understanding of how the distribution and functionality of larval breeding sites influence the evolution of malaria risk in Djilakh and suggest tailored effective prevention strategies to locally eliminate the disease.

## Methods

### Study site

**Fig. 1:**
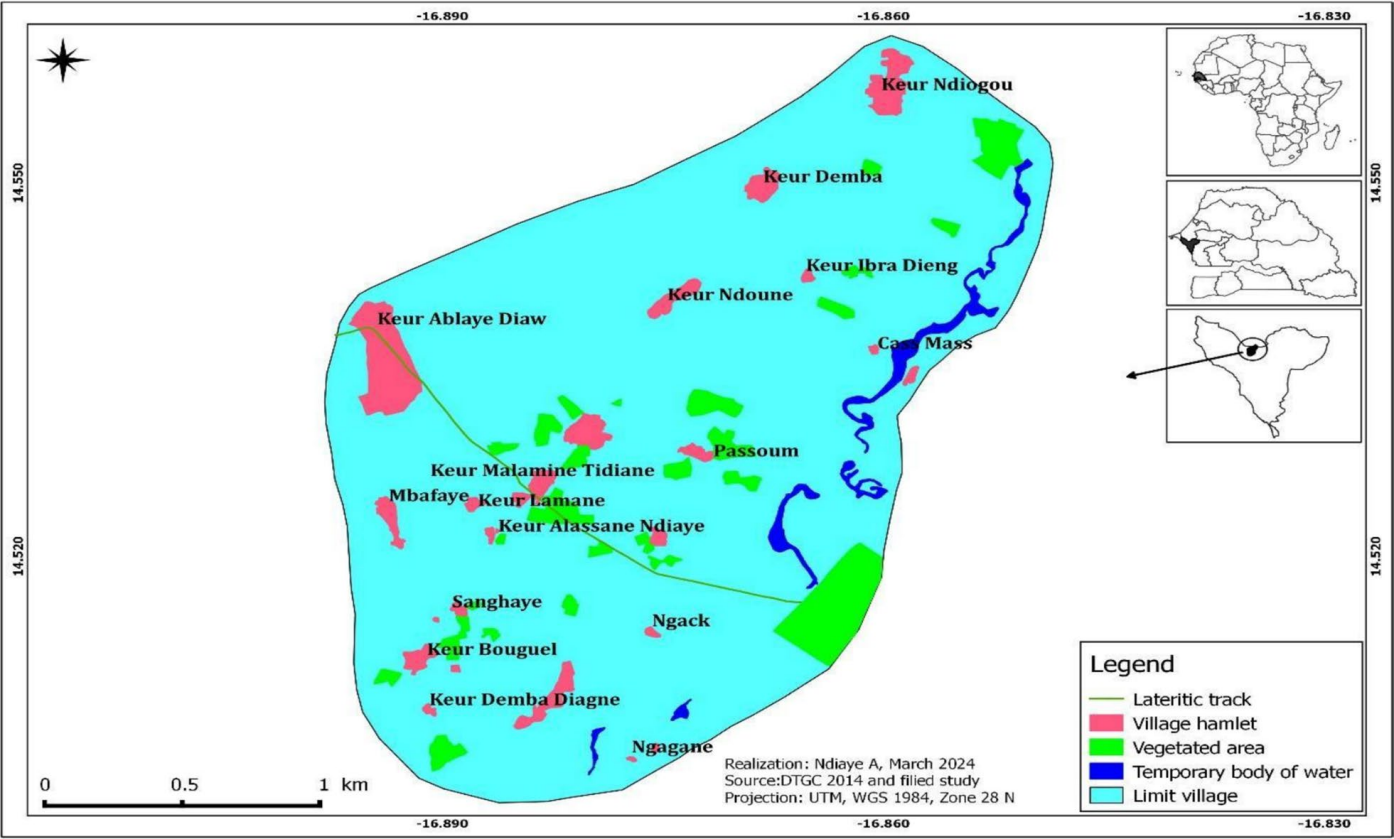
Map of the study site

Djilakh (14°31′00′′ N and 16°52′60′′ W) is constituted of ten hamlets located at the periphery of the Mbour department, specifically within the Sindia commune (Fig. 1). The landscape of Djilakh is similar to the rest of Senegal, with a relatively flat topography and few natural features. The area is characterized by low plains and rolling hills, with the highest elevation rarely exceeding 20 m. Small depressions are found in the eastern and southern parts of the village and are flooded during the rainy season. The village experiences a Saharan-Sudan climate characterized by two distinct seasons, including a prolonged dry season from November to June, and a four-month rainy season from July to October. This climatic duality shapes the rhythms of the life in the village, dictating agricultural practices and community activities throughout the year. The annual precipitation ranges from 400 to 600 mm [13], while the monthly mean temperatures are particularly high, in April, May and June, where they exceed 30 °C [14]. Most of the area is characterized by sandy soils, which are covered by tree and shrub savannah vegetation. This vegetation is mainly composed of *Acacia albida, Guiera senegalensis* and *Combretum micranthum*. Noteworthy, in the eastern and south-eastern parts of the village’s lowlands substrate is made of hydromorphic soils found on gravelly and sandy material, flooded during the rainy season up to March in some lowlands, particularly with growing grasses and herbaceous layer. The village of Djilakh was selected for the study due to the persistence of malaria transmission (more than 6 cases per year), despite the implementation of various malaria control interventions [15]. Most of the population of the study area belongs to the Serer ethnic group, who are mainly farmers [9].

## Data collection

### Malaria data collection

Malaria morbidity data were collected from the Djilakh health post. The dataset consisted of malaria cases diagnosed by rapid diagnostic test (RDT) and covers the period from 2013 to 2017. RDT is recommended as a more practical and appropriate alternative tool to microscopy since 2017 [16]. In addition to the Malaria case, additional biometric data such as the age, the sex, and the origin of the patient were collected from the health post logbook.

### Rainfall data collection

The rainfall data from 2013 to 2017 were collected from the Mbour Department weather station managed by the National Aviation and Meteorological Agency (ANACIM, https://www.anacim.sn). The monthly totals of precipitation (in millimetres) have been calculated. Rainfall data were used to assess the relationship between the rainfall and the malaria transmission dynamics for the different study periods in Djilakh village.

### Geolocalization and physico-chemical characteristics of larval habitats

The cartographic and biological data were collected over a period of 5 years (2013–2017), once every two months from June to November per year. All surface water bodies, likely to be suitable for mosquito breeding, were checked and confirmed larval habitats were characterized according to their physico-chemical and biological components, then surveyed bimonthly during the rainy and dry seasons to monitor their persistence and changes, specifically the surrounding and water bodies environmental parameters as well as the presence anopheline immature stages. The geographical coordinates of all the sites were recorded using a Garmin GPS and the proximity of human dwellings was determined using Google Earth software [17].

### Description of the breeding site’s vegetal cover, larval sampling and identification

For each surveyed breeding site, the vegetal cover was characterized, the water turbidity, the type of soil, the location of the water body to the nearest houses, the origin of the water, the depth, the size of the deposit, its positivity for *Anopheles* larvae, and other pertinent data were determined. Larvae were sampled using the dipping technique [2]. Whole water was collected for the small breeding sites, while for the bigger habitats up to 10 dips were sampled from different parts of the breeding. The whole water collected was transferred to a white container to sort and count the collected immature stage according to their genus and stage. The volume of the water collected was then measured for each breeding site. The volume of water of 1 dip (equivalent to 1/5 of litre = 200 ml) or the total volume collected from small paddles was then converted to a litre and larval density was presented as the mean density of larvae per litre. The larval density in each breeding site was calculated by instar or as the number of 3rd and 4th instar larvae of each of the anopheline species collected and reported or adjusted as the number of larvae per litre of volume for further comparison, specifically the small vs big breeding sites.

### Data analysis and processing

The logistic and Poisson regression models were used to analyze the relationships between independent variables (breeding site characteristics) and dependent variables. A preliminary analysis using a logistic regression model was run to investigate the correlation between the presence of anopheline larvae (dependent variable) in the surveyed breeding sites while the independent variables consisted of several other parameters, including the characteristics of the positive habitats, the period (month and year) of data collection. Subsequent analysis examined the correlation between the number of malaria cases and the percentage of positive breeding sites. The percentage of anopheles-positive breeding sites was calculated for every two-month period (June–July, August–September, October–November) for each collection year. A Poisson regression model was used, with the number of malaria cases as the dependent variable and the percentage of anopheles-positive breeding sites, the month and the year of collection as the independent variables. Adjusted odds ratios (for logistic regression) and incidence rate ratios (for Poisson regression) were calculated to quantify associations between variables. The presence of an odds ratio (OR) greater than 1 indicates that the variable in question has the capacity to increase the probability of the event in question. Conversely, an OR less than 1 signifies that the probability is decreased. In the event that the OR is equal to 1, it can be deduced that there is no effect on the probability. Statistical analysis was performed at a significant level of 5%. Multicollinearity was evaluated by ensuring that the variance inflation factor (VIF) was less than 5 for all independent variables. Goodness of fit was assessed using the Akaike Information Criterion (AIC) and McFadden's pseudo-R^2^, which compares the fitted model with a null model (intercept only model). McFadden's pseudo-R^2^ ranges from 0 to 1, with higher values indicating better model fit; values between 0.2 and 0.4 are generally considered acceptable [18]. The data were analyzed using Excel and by calculating cumulative means and frequencies. Furthermore, geolocated data on larval breeding sites and malaria morbidity at the hamlet level in the village of Djilakh were imported into the QGIS Desktop 2.2.0 mapping software to produce maps [19].

## Results

### Physical characteristics of larval breeding sites in Djilakh village

A total of nine natural larval breeding sites were identified in Djilakh and monitored three times per year from June to November between 2013 and 2017 (Table 1). The rainfed temporary and sunny habitats were located between 100 m and 1 km from the nearest human dwellings. The soils of the breeding sites were of clay and sandy-clay nature, accounting for 77.78% (7/9) and 22.22% (2/9), respectively (Table 1).

**Table 1:** A description of the larval habitats of *Anopheles* mosquitoes

Name of the breeding site	Type	Nature	Origin of water	Distance to dwellings (metre)	Type of soil	Sunny habitats
Ngass Ndeb	Natural	Temporary	Rain	500	Clay	Yes
Route de REVA	Natural	Temporary	Rain	1000	Clay	Yes
Faylar	Natural	Temporary	Rain	300	Clay	Yes
Cele	Natural	Temporary	Rain	400	Clay	Yes
Mboudaye	Natural	Temporary	Rain	100	Sandy-clay	Yes
Sassar	Natural	Temporary	Rain	800	Sandy-clay	Yes
Nakhane	Natural	Temporary	Rain	150	Clay	Yes
Ngass Mbaal	Natural	Temporary	Rain	300	Clay	Yes
Mbel Ngagane	Natural	Temporary	Rain	100	Clay	Yes

### Breeding site positivity and larval density seasonal variation

At the beginning of the rainy season (June-July), the positivity rate of anopheles breeding sites was 16.66%, increased to 72.22% at the middle of the rainy season (August–September) and then decreased to 55.55% toward the end of the season (October–November). The average surface of larval habitats increased parallelly from 168.05 m^2^ in June-July to 592.77 m^2^ in August–September, at the middle of the rainy season. While at the end of the rainy season (October–November), several of the surveyed sites dried up, and the average surface of those remaining decreased significantly to 287.08 m^2^ (Table 2). The highest larval densities were recorded during this latter period, with an average of 2.11 larvae per litre in 2013 and 3.22 larvae per litre in 2015. This period also recorded the most malaria cases with more than half of all cases (69%). During the same year and from August to September, the mean larval density was 0.61 larvae/litre and 0.06 larvae/litre, respectively in 2013 and 2015. From June to July 2013, a larval density of 0.04 larvae per litre was recorded, while no larvae were found for the periods of June-July 2015 and October–November 2017. In August-September2017, the breeding sites became functional once again with the larval density of 1.16 larvae per litre. The water depth remained consistently below 50 cm throughout the study period, with the exception of three sites where it exceeded 50 cm during the high-pluviometry period between August and September. The water of larval habitats was characterized by low turbidity at the beginning and end of the rainy season, becoming more in the middle of the rainy season. Vegetation was present over the time all the breeding sites, with the highest density at the middle of the rainy season (p < 0.001).

**Table 2:** Cumulative average of larval breeding site parameters during the annual study periods from 2013 to 2017

Name of the breeding site	Average water surface area (m2)	Water height (in metres)	Turbidity	Presence of *Anopheles* larvae	Presence or absence of vegetation
Period	June-July				
Ngass Ndeb	537.5	Less than 50 cm	± clear	–	Presence
Route de REVA	140	Less than 50 cm	± clear	–	Presence
Faylar	62.5	Less than 50 cm	± clear	+	Presence
Cele	327.5	Less than 50 cm	± clear	+	Presence
Mboudaye	136.25	Less than 50 cm	± clear	–	Presence
Sassar	110	Less than 50 cm	± clear	–	Presence
Nakhane	103.73	Less than 50 cm	± clear	+	Presence
Ngass Mbaal	52.5	Less than 50 cm	± clear	–	Presence
Mbel Ngagane	42.5	Less than 50 cm	± clear	+	Presence
Total average	168.05	Less than 50 cm	± clear	+	Presence
Period	August–September				
Ngass Ndeb	195	Less than 50 cm	clear	+	Presence
Route de REVA	1115	Less than 50 cm	clear	+	Presence
Faylar	387.5	More than 50 cm	clear	+	Presence
Cele	2425	More than 50 cm	clear	+	Presence
Mbpudaye	350	More than 50 cm	clear	+	Presence
Sassar	195	Less than 50 cm	clear	+	Presence
Nakhane	212.5	Less than 50 cm	clear	+	Presence
Ngass Mbaal	257.5	Less than 50 cm	clear	+	Presence
Mbel Ngagane	197.5	Less than 50 cm	clear	+	Presence
Total average	592.77	Less than 50 cm	clear	+	Presence
Periods	October–November				
Ngass Ndeb	92.5	Less than 50 cm	± clear	+	Presence
Route de REVA	500	Less than 50 cm	± clear	+	Presence
Falar	237.5	Less than 50 cm	± clear	+	Presence
Cele	1140	Less than 50 cm	± clear	+	Presence
Mboudaye	200	Less than 50 cm	± clear	+	Presence
Sassar	80	Less than 50 cm	± clear	+	Presence
Nakhane	90	Less than 50 cm	± clear	+	Presence
Ngass Mbaal	151.25	Less than 50 cm	± clear	+	Presence
Mbel Ngagane	92.5	Less than 50 cm	± clear	+	Presence
Totale average	287.08	Less than 50 cm	± clear	+	Presence

### The impact of rainfall variability on malaria transmission

**Fig. 2:**
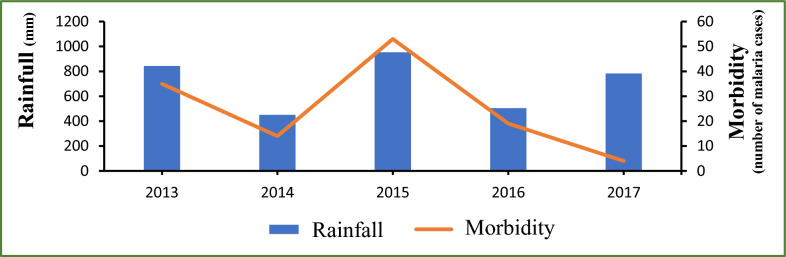
Annual variations in rainfall and the number of confirmed malaria cases in Djilakh from 2013 to 2017

**Fig. 3:**
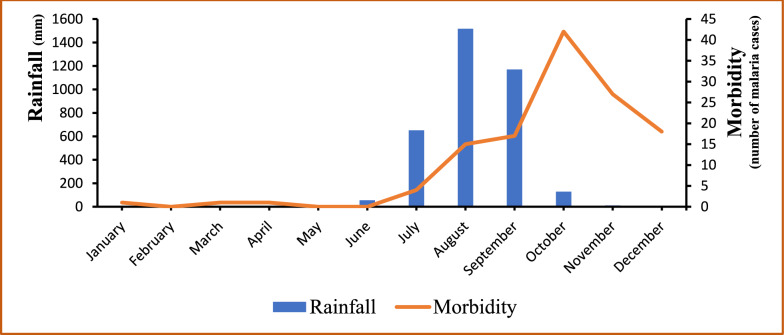
Monthly variations of rainfall and the number of confirmed malaria cases in Djilakh from 2013 to 2017

A critical examination of meteorological data and malaria morbidity indicates that malaria cases are influenced by and follow the trend of rainfall as shown in Fig. 2, with the exception of the Year 2017. The Years 2013 and 2015 corresponded with the highest levels of rainfall as well as the peaks of malaria cases; the lowest morbidities were recorded in 2014 and 2016 when the levels of rainfall were the lowest. The year 2017 was the unique exception with the lowest malaria morbidity reported while a relatively high level of rainfall was recorded in the meantime (see Fig. 2). Figure 3 illustrates a gradual increase of malaria cases from July each year, coinciding with the onset of the rainy season. The peak of incidence is observed in October, followed by a decline until December. Concurrently, the distribution of rainfall exhibits a unimodal pattern, with a peak in August, two months prior to the peak of malaria cases, recorded in October.

### Correlation between the presence of anopheline larvae and breeding site characteristics.

The association between the presence of anopheline larvae and breeding site characteristics was assessed using logistic regression (Table 3). The odds of finding *Anopheles* larvae in a given water body were significantly associated with the size of the breeding site (p < 0.05). Specifically, for each unit increase in the size of the site, the odds of finding anopheline larvae decreased by approximately 0.2%. Additionally, breeding sites located near human dwellings were strongly associated with the presence of anopheline larvae (p < 0.05). The odds of finding anopheles’ larvae in a given breeding sites located near the houses (≤ 500 m) were 27 times higher to those located above 500 m. Similarly, the association between the presence of vegetation within the breeding sites and the presence of anopheline larvae was highly significant (p < 0.001), with sites having vegetation being 63 times more likely to be positive for anopheles’ larvae compared to those without vegetation. There was also a significant seasonal effect, with breeding sites being less likely to contain anopheles’ larvae in June-July versus August–September (p < 0.05), and no significant difference between October–November and the reference period (Table 3). The model AIC is 94.83 and McFadden's pseudo-R^2^ is 0.48, indicating a good model fit compared to the null model (intercept-only model).

**Table 3:** Variation in the average larval density of breeding sites over the study periods

Name of the breeding site	Year				
	2013	2014	2015	2016	2017
Period	June-July				
Ngass Ndeb	0 larvae/litre	Not available	0 larvae/litre	Not available	Not available
Route REVA	0 larvae/litre	Not available	0 larvae/litre	Not available	Not available
Faylar	0 larvae/litre	Not available	0 larvae/litre	Not available	Not available
Cele	0.08 larvae/litre	Not available	0 larvae/litre	Not available	Not available
Mboudaye	0 larvae/litre	Not available	0 larvae/litre	Not available	Not available
Sassar	0 larvae/litre	Not available	0 larvae/litre	Not available	Not available
Nakhane	0 larvae/litre	Not available	0 larvae/litre	Not available	Not available
Ngass Mbaal	0 larvae/litre	Not available	0 larvae/litre	Not available	Not available
Mbel Ngagane	0.33 larvae/litre	Not available	0 larvae/litre	Not available	Not available
Total average	0.04 larvae/litre	Not available	0 larvae/litre	Not available	Not available
Period	August–September				
Ngass Ndeb	1 larvae/litre	Not available	1 larvae/litre	Not available	1 larvae/litre
Route REVA	0 larvae/litre	Not available	0.06 larvae/litre	Not available	0.5 larvae/litre
Falar	2 larvae/litre	Not available	0.3 larvae/litre	Not available	2 larvae/litre
Cele	0 larvae/litre	Not available	0.01 larvae/litre	Not available	3 larvae/litre
Mboudaye	1 larvae/litre	Not available	0.04 larvae/litre	Not available	1 larvae/litre
Sassar	0.5 larvae/litre	Not available	0.05 larvae/litre	Not available	1 larvae/litre
Nakhane	0 larvae/litre	Not available	0.08 larvae/litre	Not available	0 larvae/litre
Ngass Mbaal	0 larvae/litre	Not available	0.06 larvae/litre	Not available	0 larvae/litre
Mbel Ngagane	1 larvae/litre	Not available	0 larvae/litre	Not available	2 larvae/litre
Total average	0.61 larvae/litre	Not available	0.06 larvae/litre	Not available	1.16 larvae/litre
Periods	October–November				
Ngass Ndeb	2 larvae/litre	Not available	3 larvae/litre	Not available	0 larvae/litre
Route REVA	1 larvae/litre	Not available	2 larvae/litre	Not available	0 larvae/litre
Faylar	3 larvae/litre	Not available	5 larvae/litre	Not available	0 larvae/litre
Cele	5 larvae/litre	Not available	8 larvae/litre	Not available	0 larvae/litre
Mboudaye	2 larvae/litre	Not available	2 larvae/litre	Not available	0 larvae/litre
Sassar	1 larvae/litre	Not available	2 larvae/litre	Not available	0 larvae/litre
Nakhane	2 larvae/litre	Not available	1 larvae/litre	Not available	0 larvae/litre
Ngass Mbaal	1 larvae/litre	Not available	1 larvae/litre	Not available	0 larvae/litre
Mbel Ngagane	2 larvae/litre	Not available	5 larvae/litre	Not available	0 larvae/litre
Total average	2.11 larvae/litre	Not available	3.22 larvae/litre	Not available	0 larvae/litre

NB: For the years 2014 and 2016, data were not available because there was no survey of the breeding sites at these periods

### Correlation between malaria morbidity and breeding sites positivity from 2013 to 2017

The correlation between malaria morbidity and the positivity of breeding sites varied throughout the rainy season, depending on the size and stability of the breeding sites. Overall, there was a positive and highly significant correlation between malaria morbidity and the percentage of positive breeding sites (p < 0.001) (Table 4). In addition, the number of malaria cases decreased significantly over the years (p < 0.05). For each unit increase during the year, the expected number of malaria cases decreased by approximately 20.0%, after adjusting for other variables. There was also a seasonal pattern, with an important decrease in the expected number of malaria cases in June-July compared to August–September. Conversely, there was a significant increase in the expected number of malaria cases in October–November (p < 0.05), representing an increase of approximately 81.3% compared to the reference period (Table 4). The model AIC is 71.07 and McFadden's pseudo-R^2^ is 0.61, indicating a good model fit compared to the null model (intercept-only model).

**Table 4:** Correlation between presence of Anopheles larvae and different characteristics of breeding sites

Variable	Reference	Coefficient	95% CI	Standard error	Odds ratio	P-value
Intercept	–	57.153	[ − 711.234, 824.909]	386.700	–	0.883
Distance from housing	–	0.0005	[ − 0.003, 0.002]	0.001	1.000	0.642
Size of the breeding site	–	− 0.002	[ − 0.004, 0.0005]	0.001	0.998	0.019
Breeding site near housings	Breeding site far from housings	3.296	[0.895, 6.055]	1.292	27.006	0.011
Presence of the vegetation	Absence of vegetation	4.147	[2.284, 6.602]	1.078	63.214	0.0001
Height over 50 cm	Height below 50 cm	2.393	[0.099, 6.077]	1.422	10.950	0.092
Sandy-clay soils	Clay soils	− 0.612	[ − 2.218, 0.899]	0.782	0.542	0.433
June-July	August–September	− 1.700	[ − 3.311, − 0.241]	0.773	0.183	0.028
October–November		1.262	[ − 0.384, 3.361]	0.915	3.531	0.168
Year	–	− 0.030	[ − 0.410, 0.351]	0.192	0.971	0.877

Results of logistic regression: regression coefficients, standard error, odds ratio and p-value. CI stands for confidence intervals. Model AIC: 94.83 and McFadden’s pseudo-R^2^: 0.48

**Fig. 4:**
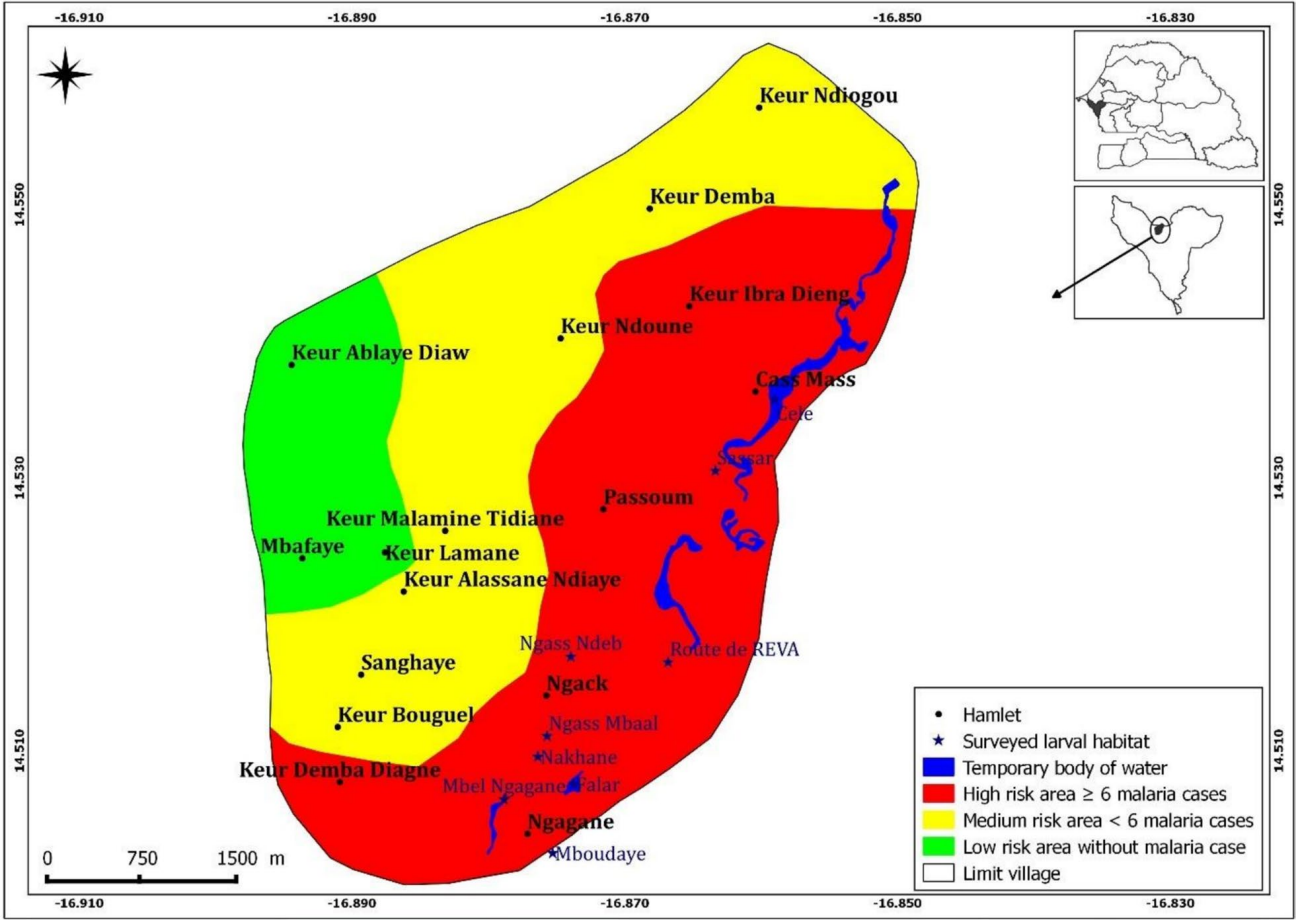
Malaria risk map of Djilakh

A significant high number of malaria cases (82.4%, 103/125) was recorded in the hamlets at the vicinity of the larval habitats, approximately less than 500 m apart from them; followed by those located between 500 to 1000 m from the breeding sites (17.6%, 22/125). Noteworthy, villages at the most remote location, above 1000 m from the functioning breeding sites, were free of malaria during the study period. Overall, the number of malaria cases decreased from the eastern part of the village, where the larval habitats were concentrated, to the western part, where no larval habitats were observed. A close look at the soil’s composition showed that with the exception of the eastern part of the village, the soil of the rest of the study area is made of tropical ferruginous soils with minimal or no leaching soils (known as "dior" soils). The so-called "dior" soil is of sandy texture and thus is highly permeable, leading to its low capacity for surface water retention and more penetration toward the phreatic water nappes. Given that the risk of malaria transmission is likely associated with the presence of the natural breeding sites identified in the area, the morbidity data allowed the study village to be stratified into three risk zones, considering the position of the larval habitats in relation to the study site (Fig. 4).

## Discussion

A more comprehensive understanding of malaria and its transmission dynamics can be achieved by integrating epidemiological to environmental data. The persistence of residual malaria transmission within an overall low endemicity area is largely influenced by climatic variables, particularly rainfall and environmental conditions, and has been shown to have a pronounced effect on the biology of the vector and the parasite it harbors in some specific regions.

In the study area, all larval breeding sites were temporary rainfed and sunny pools with vegetation. All the functioning larval habitats were located toward the eastern part of the study village and characterized by hydromorphic soils with a fairly high inclusion of clay soils, which increased their water retention capacity and thus creating numerous surface water bodies suitable for the anopheline larvae development. These findings are consistent with those of previous studies, which indicate that various *Anopheles* species use a range of water sources as breeding sites, including stagnant sunny residual surface water pools, sometime with emerged vegetation, and/or in some cases brackish water [14, 20]. The formation of anopheles’ larval habitats is likely to be largely determined by the nature of the soil [21]. The type of soil and its related water-retention capacity seems to have contributed to the observed residual transmission in the study village. In particular, hydromorphic and halomorphic soils are an important factor for the development of *An. arabiensis* and *An. melas* larvae, given their capacity to retain surface water when those are scarce in the surrounding areas, and specifically brackish breeding sites suitable for *An. melas* larvae described as an important focal malaria vector in the study area [14, 22].

The trend of malaria in a given place and period is influenced by the variation and periodicity of rainfall, which determines the frequency of tidal movement of some deltaic water system and thus the flooding of breeding sites. The study showed that the peak in morbidity occurs two months after the rainy season, likely explained by the instability and washout of breeding sites during the high rain intensity period in August–September. Which is consistent with previous findings from this region, indicating a lag of approximately one to two months between the peak of rainfall and the highest incidence of malaria-related incidence [23], once the rains stop allowing more stable and productive breeding sites.

The results of this study indicate a positive correlation between annual rainfall variability and malaria morbidity. An increase in rainfall leads to the proliferation and abundance of natural breeding sites, which in turn favors the increase of vector density and consequently malaria transmission as previously shown from previous studies in 2010, which demonstrated that rainfall exerts a significant influence on malaria incidence, which remains high during years characterized by abundant rainfall [24]. However, factors such as the availability of mosquito breeding sites, human behaviour (e.g. health care seeking, use of mosquito nets) and control interventions such as seasonal malaria chemoprevention (SMC) and indoor residual spraying (IRS) can also influence the occurrence of cases [5, 25]. In 2017, there was a notable increase in rainfall, but a notable decline of the confirmed malaria cases. This could be explained by the low larval density in the middle of the season due to the high rainfall and the observed low presence and productivity of *Anopheles* breeding sites toward the end of the rainy season (October–November), which used to be the period of high vector densities and thus high transmission period in the study area.

The increase in precipitation during the middle of the rainy season (August–September) results in an elevated number and surface water bodies, thus providing anopheles mosquitoes more breeding sites. However, the highest larval densities were observed at the end of the rainy season (October–November), corresponding to the decline and more spaced rainfalls, allowing breeding sites no longer washed out to become more stable and productive, thereby facilitating the proliferation of vector populations. Similar observations were made by Stefani [26], who showed that the low intensity and low frequency of rainfall following the peak of the season result in the proliferation of more stable breeding sites, thus leading to high vector densities. While heavy rainfall can result in the washout out of breeding sites, thereby reducing larval populations.

The results indicate that hamlets located within 500 m of the breeding site are more exposed to malaria risk than those situated at a distance greater than 1000 m. Malaria cases was much higher in hamlets that were closest to anopheles larval breeding sites, decreasing progressively with distance to those, as previously shown by Brissy et al*.* [27], who showed that malaria risk depends on the proximity of vector breeding sites.

The results demonstrated the correlation between environmental and entomological factors in relation to malaria morbidity in Djilakh. The utilization of these data could assist in identifying the factors associated with the specific epidemic profile for this malaria hotspots village. Furthermore, it could facilitate the analysis of the dynamic characteristics of the factors associated with temporal variations in transmission within remaining malaria hotspots.

Despite the important findings, this study has some limitations. Firstly, the malaria epidemiological data obtained from the Djilakh health post was limited to only the number of malaria cases confirmed by rapid diagnostic tests. Therefore, the analysis was limited to the morbidity data, with the knowledge of self-medication or use traditional medicine thus low care-seeking behaviour among sick villagers, as often reported in the country. This information was not taken into account, and it is recognized that this was a significant limitation as it would have provided a more detailed and complementary view of this work. Secondly, the cartographic and biological datasets utilized for the identification and characterization of the various breeding sites were incomplete, with one such dataset lacking data for the years 2014 and 2016 due to resource constraints, thus preventing the calculation of certain parameters, including larval densities for these years. The missing data for this period has been excluded from the models. The authors chose not to interpolate the missing values using the data of the other years, because this could introduce a bias and distort the relationship with the variable year.

## Conclusion

Despite being preliminary, the findings from this study represent step towards a more comprehensive understanding of the environmental factors influencing the persistence of residual malaria transmission in Senegalese hotspot villages, notwithstanding the deployment of various vector control measures. This study illustrates the value of a geographical approach in characterizing the environmental conditions favoring vector development and human-vector interactions within the study area. A more comprehensive understanding of the environmental factors that contribute to malaria transmission in endemic areas will inform the development of more effective strategies to eliminate the disease. These strategies must consider that the environmental factors within these regions is often heterogeneous. In the future, a more intelligent targeting of interventions could be envisaged, by accounting also epidemiological, geographical, entomological and environmental realities.

## Data Availability

No datasets were generated or analysed during the current study.
